# Molecular determinants of picornavirus 3C protease binding to phosphoinositide-enriched lipid membranes

**DOI:** 10.1016/j.jbc.2025.110543

**Published:** 2025-07-30

**Authors:** Jie Yu, Dennis S. Winston, David D. Boehr

**Affiliations:** Department of Chemistry, The Pennsylvania State University, University Park, Pennsylvania, USA

**Keywords:** picornavirus, poliovirus, peripheral membrane-binding protein, lipid nanodisc, phosphoinositide, 3C protease, paramagnetic relaxation enhancement

## Abstract

Picornavirus infection leads to the production of new membranous structures that are enriched in phosphoinositide lipids. The multifunctional 3C protease interacts with these phosphoinositide-enriched membranes. We performed paramagnetic relaxation enhancement NMR experiments to gain insight into the molecular determinants driving these interactions. We found that 3C interacts with lipid membranes through both its positively charged N-terminal helix and the 80 to 90’s region, previously proposed to interact with RNA. Experiments using different pH and salt concentrations suggest that the interaction is primarily electrostatically driven. The optimized lipid membrane systems developed here provides molecular insights into the 3C-membrane interactions and can be leveraged to study peripheral protein-membrane interactions of other viral proteins.

Picornaviruses are positive-strand RNA viruses and include many important human and animal pathogens (1). Poliovirus (PV) is the most extensively studied virus in this order and is an important model system. PV induces temporally and structurally distinct membranous structures (“organelles”) for genome replication and virus assembly in which the transition from replication-to-assembly organelles is likely set by the rate of P3 polyprotein processing and a consequent increase in 3CD concentration (2). The 3CD protein, comprised of 3C protease and 3D RNA-dependent RNA polymerase domains, has been shown to hijack the GBF1-Arf1-PI4KB pathway, induce the synthesis of phosphatidylinositol-4-phosphate (PI4P) and phosphatidylinositol-4,5-bisphosphate (PI(4,5)P_2_), as well as phosphatidylcholine (PC) and promote the proliferation of membranes in the perinuclear region in cells (2, 3, 4).

Previous studies have suggested direct interactions between phosphoinositides (PIPs) and PV proteins 3C, 3D, and 3CD. The 3D protein was known to associate with membranes and catalyze the synthesis of viral RNA on these membranes, and it was found to bind soluble PI4P using lipid strips (5) and PI4P in a membrane context on supported lipid bilayers (6) or vesicles (7). The 3CD and 3C proteins have also been shown to bind PIPs in a membrane context (3, 8). However, these studies have provided limited molecular-level understanding of the mechanisms driving the interactions between these PV proteins and membranes.

Membrane interactions may affect PV protein function. For example, the proteolytic activity of Senecavirus A 3C protease has been suggested to be allosterically regulated by phospholipids cardiolipin, PI4P, and sulfatide (9). Cholesterol levels have also been found to increase upon infection by PV and coxsackievirus B3 (CVB3), and disruption of cholesterol in membranes results in increased proteolysis of 3CD (4). These studies suggest that the expression levels and functions of viral proteins may be regulated by membrane lipid composition. For some other membrane-binding proteins, protein–membrane interactions have likewise been shown to allosterically regulate their function (10, 11, 12, 13, 14). Modulation of protein function upon membrane binding could serve as a way for further regulation of the replication cycle and as a way for PV to expand the functionality of its limited proteome.

In the current research, we have developed and optimized lipid membrane systems that are amenable to nuclear magnetic resonance (NMR) spectroscopy studies to provide molecular insights into the PV 3C-membrane interactions. Specifically, we conducted solution-state NMR studies using lipid nanodiscs enriched in PIPs and embedded with a special lipid capable of chelating the paramagnetic gadolinium ion (Gd^3+^). Paramagnetic relaxation enhancement (PRE) resulting from the close association of 3C amino acid residues with the lipid nanodisc allowed us to better map the membrane-binding site of 3C and compare these results against previous molecular dynamics (MD) simulations and NMR experiments with soluble PIPs (8). Our results indicate that 3C-membrane binding is stronger under lower salt concentration and lower pH conditions, likely due to enhanced electrostatic attraction. Hijacking of host membranes for viral replication or assembly is common to other RNA viruses (15, 16, 17). Also, viral protein–membrane interactions are known to play important roles in the viral life cycle. Influenza A virus matrix protein 1 (M1), Ebola matrix protein VP40, and HIV matrix protein (MA) all bind to membranes with negatively charged lipids, with influenza A virus M1 binding to phosphatidyl serine, Ebola VP40 binding to PS and PI(4,5)P_2_, and HIV MA binding to PI(4,5)P_2_. These viral protein-membrane interactions facilitate viral assembly and budding (18, 19, 20). As such, methods developed here can be leveraged towards understanding interactions for other peripherally-associated viral membrane proteins, which can be targeted with antiviral therapies.

## Results

### Solution-state NMR studies reveal membrane-binding sites on PV 3C

**Figure 1:**
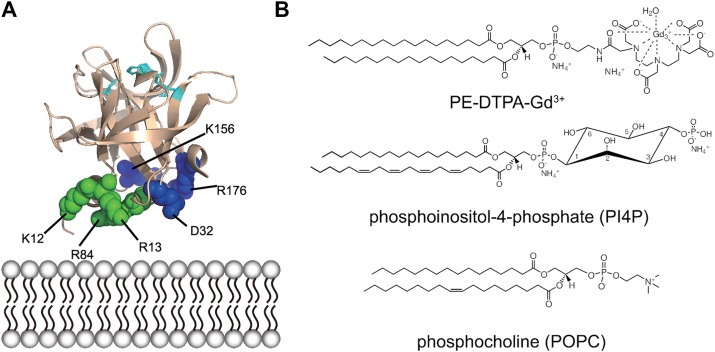
**Structure of poliovirus 3C and lipids used in nanodiscs.***A*, structure of poliovirus 3C (pdb id: 1l1n) with the proposed PI4P binding surface shown as spheres and active site residues H40, E71, and C147 shown in *cyan*. The PI4P-binding residues identified from docking and previous NMR experiments (K12, R13, and R84) are shown in *green*. The additional PI4P-binding residues predicted from molecular dynamics simulations (D32, K156, R176) are shown in *blue*. *B*, chemical structures of selected phospholipids.

To gain atomic-level insights into the interactions between 3C and phosphoinositide-enriched lipid bilayers, we conducted solution-state NMR studies using lipid nanodiscs to present these PIPs in a lipid bilayer context. These nanodiscs also have incorporated a special lipid (phosphorylethanolamine - diethylenetriaminepentaacetic acid, PE-DTPA) that chelates the paramagnetic gadolinium ion (Gd^3+^) (Fig. 1). This design allowed for PRE NMR-based experiments, which can provide distance-dependent information due to increased relaxation of nuclei that are in close proximity to paramagnetic ions (21). That is, when protein interacts with the lipid nanodiscs, resonances belonging to amino acid residues near the lipid membrane will be lower in intensity in the presence of paramagnetic nanodiscs compared to in the presence of diamagnetic nanodiscs (*i.e.* no PE-DTPA-Gd^3+^ present) due to increased transverse relaxation.

**Figure 2:**
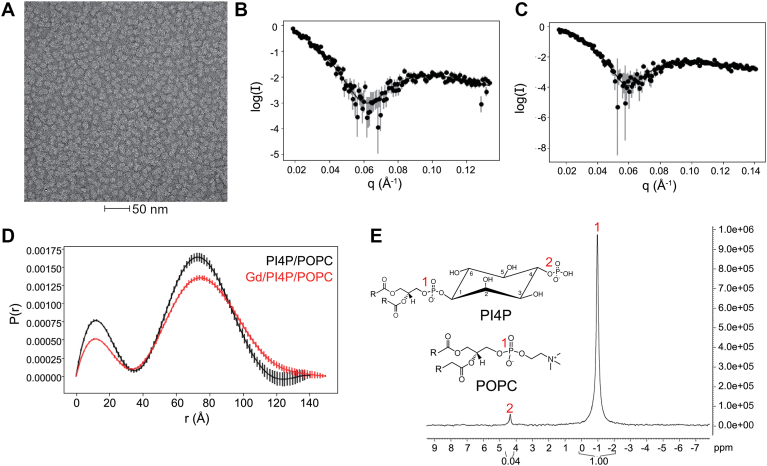
**Characterization of lipid nanodiscs.***A*, transmission electron microscopy image of POPC nanodiscs purified with FPLC. *B*, raw solvent-corrected small-angle X-ray scattering data for nanodiscs containing 7.5% PI4P, 92.5% POPC (*black*). *C*, raw solvent-corrected scattering data for nanodiscs containing 2.5% PE-DTPA-Gd^3+^,7.5% PI4P, 90% POPC. *D,* pairwise distance distributions calculated using the autoGNOM function in Primus (47). Error bars represent errors from the fit. *E*, ^31^P NMR spectrum of 4% PI4P, 96% POPC nanodiscs, integrated using Bruker Topspin.

More specifically, we generated nanodiscs with 16:0 to 18:1 phosphocholine (POPC), PI4P and PE-DTPA-Gd^3+^ using previously described procedures (22). The size, shape and composition of the lipid nanodiscs was verified using transmission electron microscopy (TEM), ^31^P NMR and small angle x-ray scattering (SAXS) (Figs. S1 and S2). The nanodiscs were purified by fast protein liquid chromatography (FPLC)-size exclusion chromatography (SEC) (Fig. S1). Based on TEM, they appeared to be homogenous and have a diameter around 10 nm, as expected (Fig. 2*A*). From SAXS, the pairwise distance distribution functions for POPC nanodiscs containing 7.5 mol % PI4P and 7.5 mol % PI4P, 2.5 mol % PE-DTPA-Gd^3+^ were bimodal, consistent with the majority of the electron density coming from the scaffold protein that encircles the lipid bilayer (Fig. 2*D*). The scattering profiles and pairwise distance distribution functions appear very similar between the two nanodisc samples (Fig. 2, *B* and *C*), indicating that the nanodisc size and shape does not change substantially when Gd^3+^ is incorporated. The difference in scattering intensity between the two nanodisc samples is likely due to differences in concentration. To confirm the lipid composition of the nanodiscs, ^31^P NMR spectroscopy was performed on 4% PI4P, 96% POPC nanodiscs. The phosphate that connects the inositol headgroup to the rest of the lipid has the same chemical shift in POPC and PI4P and is expected to account for most of the ^31^P in the spectrum, while the phosphate in the PI4P headgroup is shifted downfield (23). Integration of the NMR peaks confirms that around 4% of the phosphorous signal is from PI4P (Fig. 2*E*). The broad linewidths in the NMR spectrum (∼0.2 ppm or ∼70 Hz) confirm that the lipids are part of the nanodisc, as the rotational correlation time for a ∼5 nm radius nanodisc should be much larger than that of free lipids (*i.e.* for small molecules, we expect linewidths less than ∼0.01 ppm or ∼5 Hz) or micelles.

**Figure 3:**
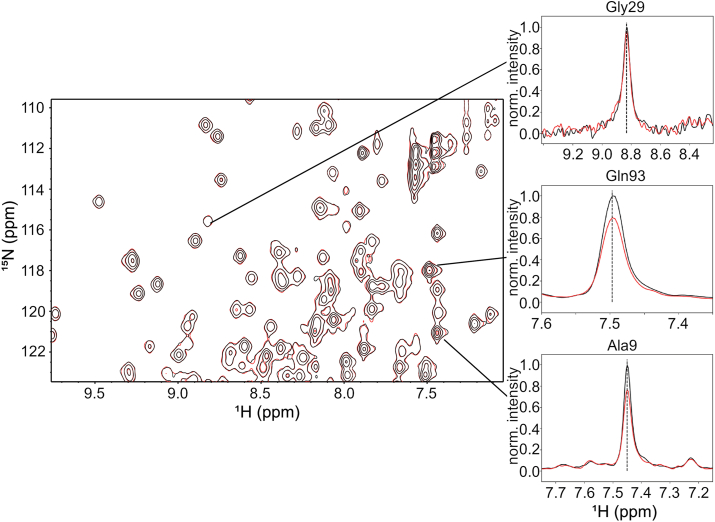
**PRE-based NMR experiments were used to identify the lipid membrane binding sites of PV 3C.** The *red* spectrum represent the 3C resonances in the presence of paramagnetically-labeled lipid nanodisc (5 μM 2.5% PE-DTPA-Gd^3+^, 7.5% PI4P, 90% POPC nanodiscs, 110 μM 3C in 20 mM HEPES pH 7, 60 mM NaCl, 10% D_2_O, 298 K), and the *black* spectrum represent the 3C resonances in the presence of the lipid nanodisc without paramagnetic label (5 μM 7.5% PI4P, 92.5% POPC nanodiscs, 110 μM 3C in 20 mM HEPES pH 7, 60 mM NaCl, 10% D_2_O, 298 K) from SOFAST ^1^H-^15^N HMQC spectra.

For the PRE-based NMR experiments, the intensity of each resonance from ^1^H-^15^N band-selective optimized flip-angle short transient (SOFAST) - heteronuclear multiple quantum coherence (HMQC) NMR spectra was compared between 3C with “paramagnetic” nanodiscs (2.5% PE-DTPA-Gd^3+^, 7.5% PI4P, 90% POPC) and “diamagnetic” nanodiscs (7.5% PI4P, 92.5% POPC) (Fig. 3). As negative controls, spectra were collected for 3C with 97.5% POPC, 2.5% PE-DTPA-Gd^3+^ or 100% POPC nanodiscs (Fig. S2). It should be noted that accurate determination of distances from PRE experiments, while theoretically possible, is very challenging to achieve experimentally (24). Due to limited sample stability, we were not able to determine the intensity for the same sample at two different delay times as recommended (24). As a result, we could not determine the distances between the 3C amide protons and the membrane accurately. Nonetheless, our experiments provide a qualitative view of 3C interactions with the lipid nanodiscs.

**Figure 4:**
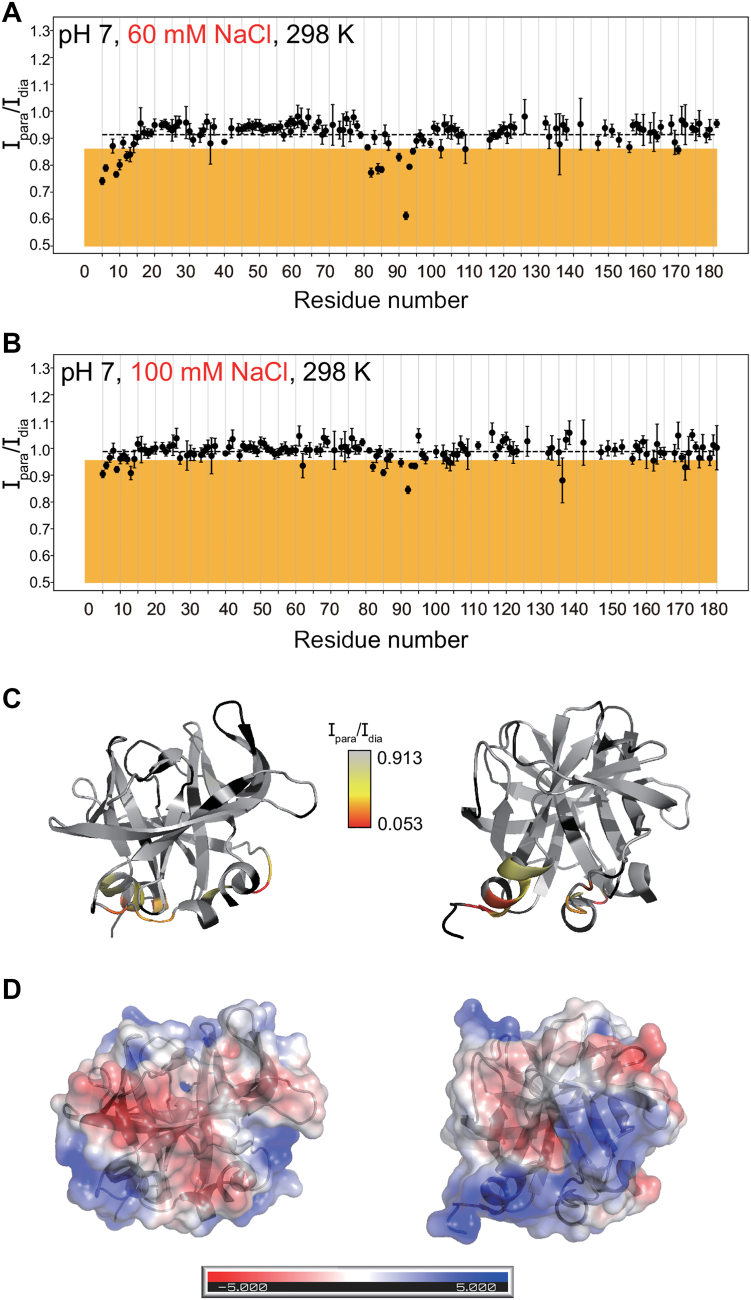
**The PV 3C protein interacts with PI4P-enriched nanodiscs more strongly at lower salt concentrations.***A* and *B*, intensity of resonances for paramagnetic sample (*A*: 5 μM 2.5% PE-DTPA-Gd^3+^, 7.5% PI4P, 90% POPC nanodiscs, 110 μM 3C in 20 mM HEPES pH 7, 60 mM NaCl, 10% D_2_O, 298 K, *B*: 5 μM 2.5% PE-DTPA-Gd^3+^, 7.5% PI4P, 90% POPC nanodiscs, 110 μM 3C in 20 mM HEPES pH 7, 100 mM NaCl, 10% D_2_O, 298 K) divided intensity for diamagnetic sample (*A*: 5 μM 7.5% PI4P, 92.5% POPC nanodiscs, 110 μM 3C in 20 mM HEPES pH 7, 60 mM NaCl, 10% D_2_O, 298 K, *B*: 5 μM 7.5% PI4P, 92.5% POPC nanodiscs, 110 μM 3C in 20 mM HEPES pH 7, 100 mM NaCl, 10% D_2_O, 298 K) from SOFAST ^1^H-^15^N HMQC spectra. The mean value is shown by a dashed line and the *orange* background shows residues with intensity ratio one standard deviation below the mean. Error bars reflect noise levels of the spectra, according to the procedure detailed in the Experimental Procedures. *C*, intensity ratios from *panelA* plotted onto the 3-dimensional structure of poliovirus 3C (pdb id: 1l1n), shown in two orientations. Residues with no data available are shown in *black*. *D*, electrostatic potential map of the surface area of PV 3C protein. Positive potential is highlighted in *blue*, negative one in *red*, neutral regions are shown in *white*. The protein backbone is colored in *dark grey*.

**Figure 5:**
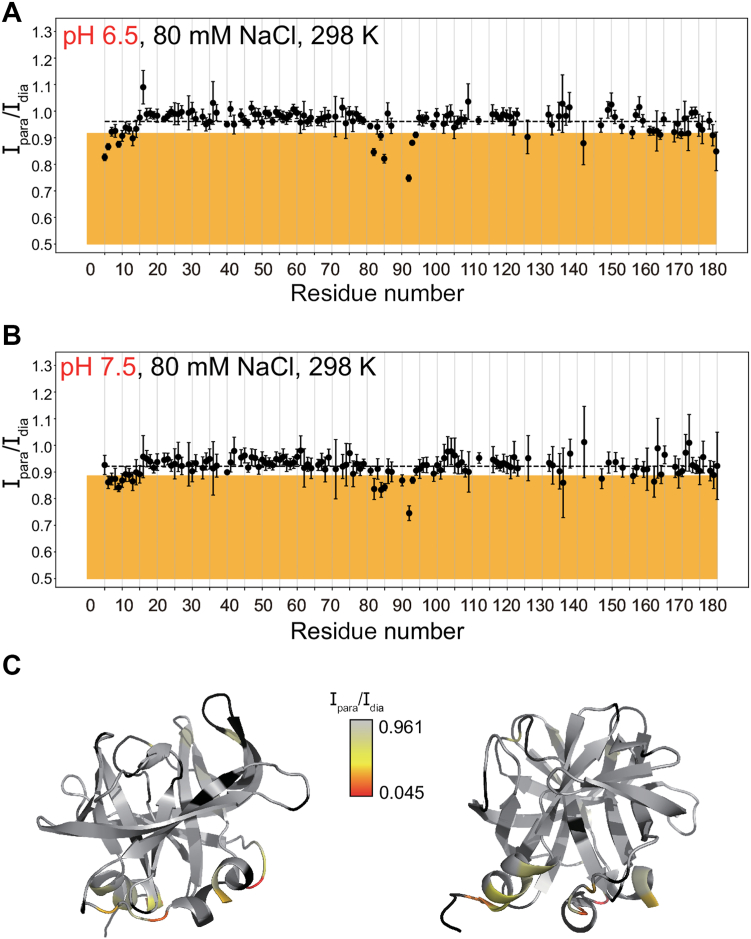
**The PV 3C protein interacts with PI4P-enriched nanodisc more strongly at lower pH conditions.***A* and *B*, intensity of resonances for paramagnetic sample (*A*: 5 μM 2.5% PE-DTPA-Gd^3+^, 7.5% PI4P, 90% POPC nanodiscs, 110 μM 3C in 20 mM HEPES pH 6.5, 80 mM NaCl, 10% D_2_O, 298 K, *B*: 5 μM 2.5% PE-DTPA-Gd^3+^, 7.5% PI4P, 90% POPC nanodiscs, 110 μM 3C in 20 mM HEPES pH 7.5, 80 mM NaCl, 10% D_2_O, 298 K) divided intensity for diamagnetic sample (*A*: 5 μM 7.5% PI4P, 92.5% POPC nanodiscs, 110 μM 3C in 20 mM HEPES pH 6.5, 80 mM NaCl, 10% D_2_O, 298 K, *B*: 5 μM 7.5% PI4P, 92.5% POPC nanodiscs, 110 μM 3C in 20 mM HEPES pH 7.5, 80 mM NaCl, 10% D_2_O, 298 K) from SOFAST ^1^H-^15^N HMQC spectra. The mean value is shown by a dashed line, and the *orange* background shows residues with an intensity ratio one standard deviation below the mean. Error bars reflect noise levels of the spectra, according to the procedure detailed in the Experimental Procedures. *C*, intensity ratios from *panelA* plotted onto the three-dimensional structure of poliovirus 3C (pdb id: 1l1n), shown in two orientations. Residues with no data available are shown in *black*.

**Figure 6:**
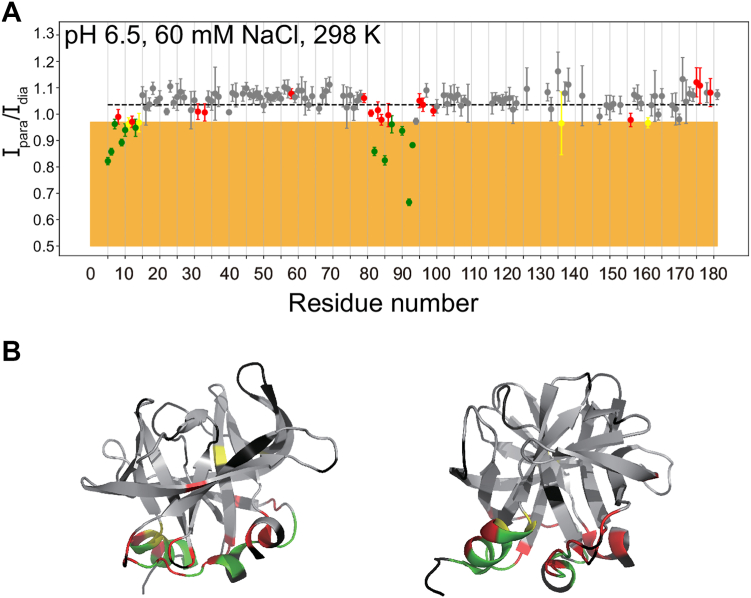
**The experimental NMR results largely agree with previous MD simulations of the 3C-membrane interactions.***A*, PRE data at conditions involving pH 6.5, 60 mM NaCl, 298 K with points colored based on agreement between PRE data and MD simulations (taken from ref (8)). Residues were considered close to the membrane in the PRE experiment if they had Ipara/Idia less than one standard deviation below the mean, and close to the membrane in the MD simulation if they were within 5 Å of the membrane during the last 300 ns of the 400 ns MD simulation. Residues were colored as follows. *Green*: close to the membrane in both PRE and MD. *Red*: close to the membrane in MD but not PRE. *Yellow*: Close to the membrane in PRE but not MD. *Gray*: Not close to the membrane in either PRE or MD. *Black*: no PRE data. *B*, structure of 3C colored with the same scheme as *panelA* (pdb 1l1n). Error bars reflect noise levels of NMR spectra.

To identify amino acid residues close to the membrane surface in the 3C-nanodisc complex, we compared ^1^H-^15^N resonance intensity ratios (I_para_/I_dia_) between paramagnetic (I_para_, 3C in the presence of PE-GTPA-Gd^3+^ labeled nanodiscs) and diamagnetic (I_dia_, 3C in the presence of nanodiscs without PE-GTPA-Gd^3+^), highlighting those resonances (in the orange region) that fall one standard deviation below the mean (Figure 4, Figure 5, Figure 6). The corresponding residues include those located at the N-terminal helix, around the previously identified RNA binding region (residues 80–95) and near the C-terminus (Figure 4, Figure 5, Figure 6). The binding interface identified here by PRE overlaps with the RNA binding site (25) and is consistent with the previously proposed phosphoinositide-binding site (8). For negative control experiments using nanodiscs without PI4P, PRE effects were not observed (Fig. S2), which indicates the importance of PI4P for 3C membrane binding.

### Lower salt concentrations lead to stronger 3C-membrane interactions

To gain insight into the nature of the protein-membrane interactions, PRE experiments were also performed under different pH conditions (6.5, 7, and 7.5) and salt concentrations (60, 80, and 100 mM NaCl) (Figure 4, Figure 5 and S3). At the lower NaCl concentration (60 mM), there were larger decreases in peak intensities at all pH tested (Figs. 4 and S3), suggesting 3C interacts more with the lipid nanodisc at lower salt concentrations. Stronger binding at lower salt concentration is likely due to a stronger surface charge, which strengthens the electrostatic attraction with the more positively charged protein (Fig. 4*D*) and negatively charged membrane. However, it is noted that some of the residues associated with larger PRE are predicted to be negatively charged (*e.g.* D5, D85), which suggests that the overall charge is more important and/or that other specific interactions are involved (*e.g.* H-bond interactions).

### Lower pH conditions also lead to stronger 3C-membrane interactions

Similarly, PRE effects were more pronounced at pH 6.5 compared to pH 7.5 at all salt concentrations (Figs. 5 and S3). Again, this finding is consistent with electrostatic interactions being important in the 3C-membrane interactions with a more positively charged 3C at lower pH conditions interacting more favorably with the negatively charged membrane.

It should be noted that we also performed experiments at two different temperatures, 293 K and 298 K, with quite similar results (Fig. S4). Unfortunately, sample and instrument limitations prevented a larger range of temperatures from being tested, and thus little can be gleaned about the relative importance of enthalpic and entropic factors governing the binding interaction.

### The binding mode of 3C to the lipid nanodisc largely agrees with MD simulations

We also compared the experimental NMR results (*i.e.* conditions involving pH 6.5, 60 mM NaCl, 298 K) to previously performed MD simulations (8) (Fig. 6). To make this comparison, we considered residues close to the membrane in the MD simulation to be those that are within 5 Å of the membrane during the last 300 ns of the 400 ns simulation. If the experiment and simulation agree, we would expect residues close to the membrane in the simulation to have strong PRE in the NMR experiment.

For most residues with strong PRE, those residues were within 5 Å of the membrane in the MD simulation (green in Fig. 6). However, several residues with strong PRE were not within 5 Å of the membrane in the simulation (yellow in Fig. 6), and about half of the residues that were within 5 Å of the membrane in the simulation did not experience strong PRE (red in Fig. 6). It should be noted that most of these residues are very close to residues that did experience strong PRE effects so differences may be minor. Overall, there is good agreement between the PRE data and the MD simulations, helping to experimentally confirm the previous MD simulations.

### Comparison of the membrane binding affinity of 3C variants with and without the C-terminal hexahistidine tag

Previous NMR studies of 3C binding to soluble PIPs used a construct with a hexahistidine tag attached by a GSSG linker to the C-terminus (8). Our preliminary studies had indicated that the His-tagged version of 3C bound to soluble PIPs with higher affinity than 3C without the His-tag (*i.e.,* the version used in this study) (Chan and Boehr, *unpublished*). In the PRE experiments, the results with His-tagged and untagged versions of the protein were very similar (Fig. S5), although the PRE results may not reflect differences in binding affinity.

We also evaluated membrane binding using a supported lipid bilayer binding assay as we have done before (8). Using a Langmuir isotherm with 1:1 binding stoichiometry, dissociation constants were determined to be 2.1 ± 0.9 μM and 0.41 ± 0.09 μM for the untagged and His-tagged versions, respectively (Fig. S6), suggesting that the C-terminal His-tag increases the binding affinity of 3C for membranes in the context of this assay. We note that binding is likely weaker to the lipid nanodiscs themselves as the PRE experiments are dependent on a fast-exchanging system.

## Discussion

Lipid membrane reorganization occurs in cells upon infection by positive-strand RNA viruses; several picornavirus proteins have been shown to interact with these membrane structures, either as integral membrane proteins (*e.g.,* 3A) or as peripheral, surface-interacting proteins (*e.g.* 3C, 3D and 3CD). These membrane interactions may lead to changes in protein conformations, protein interactions and other functions. The interaction of PV 3C with phosphoinositide-containing lipid bilayers has previously been studied by lipid strip binding assays, NMR studies with soluble PIP analogs, molecular docking, and MD simulations (8). Here we have provided, for the first time, experimental data on the mode of membrane binding of poliovirus 3C in a lipid bilayer context. The 3C protease interacts with phosphoinositide-containing membranes mainly through its positively charged N-terminal helix and the RNA binding region (residues 80–95). This provides a contiguous surface allowing 3C to interact with the membrane. Multiple-sequence alignment of enteroviral 3C proteins shows that most of the residues identified by PRE experiments are highly conserved (Fig. S7); this amino acid sequence is unique compared to other PIP-binding proteins.

We note that there are some differences compared to previous NMR studies with His-tagged 3C and soluble PI4P (8) (Fig. S8). Large changes occurred in both experiments at the N-terminal helix, especially for R13, the region around R84, and T180. In the titration using soluble PI4P, some residues (*i.e.* E45, T132, A133, G163 and H168) had substantial chemical shift perturbations but did not show significant decreases in peak intensity upon interaction with the PRE-labeled nanodiscs. It is noted that chemical shift perturbations can result from “indirect” effects (*e.g.* ligand-induced conformational change), whereas the PRE-based experiments depend only on proximity to the paramagnetic substance and are a more direct way of measuring binding effects. In addition, presentation of the PI4P lipids in a membrane context may change the ability of some residues to interact with these lipids.

The PRE experiments show that the 3C-membrane binding is stronger at lower ionic strength. The strong dependence of peripheral membrane protein-membrane binding on salt concentration has been reported for other systems (26). Weaker binding at higher salt concentration is likely due to a weaker surface charge. At higher salt concentration, the absolute value of the electrostatic potential is lower at the surface and decreases more rapidly as a function of distance from the surface, weakening the electrostatic attraction between the positively charged protein and the negatively charged membrane. It should be noted that the ionic strength in PRE experiments is below physiological, although ∼100 mM NaCl or KCl is commonly used in biophysical studies on protein-membrane interactions, especially in NMR studies (23, 27, 28, 29, 30, 31).

It has also been shown that the interaction between a protein and phosphoinositide-containing membranes can be modulated by pH. For example, binding of Osh1 to PI4P in the yeast trans-Golgi network (TGN) is dependent on intracellular pH (32, 33). Other research reported on a similar mechanism of the interaction between Opi1 and phosphatidic acid (34, 35). “Electrostatic/hydrogen bond switch mechanism” has been used to describe how these proteins bind preferentially to deprotonated phosphatidic acid and PI4P (32, 34, 36): increased charge on lipid strengthens both the electrostatic and hydrogen bonding interactions with the protein. However, our study shows a different pattern of how pH affects protein-membrane interaction; that is, studies reported here suggest that the 3C-membrane interaction is stronger at lower pH conditions. The isoelectric point of 3C is 8.2 (8), so 3C is overall positively charged in our PRE experiments; the phosphates in lipid head groups have pK_a_’s ∼1 to 2. However, there are histidine residues within (*i.e.* H89) or near (*i.e.* H31) the proposed membrane binding site, which likely have pK_a_ values ∼6 to 7, and may help to modulate the pH response. Consistent with this idea, comparisons of the NMR spectra at pH 6.5, 7.0 and 7.5 (Fig. S9) show small chemical shift changes for these and nearby residues (*e.g.* F179 makes contact with H31, both near the membrane-binding site). However, these changes are localized and there does not appear to be larger pH-induced conformational changes, as has been observed for some other proteins that bind to membranes stronger at lower pH (37, 38). Mutagenesis of these residues could serve to evaluate both the binding mechanism and any biological relevance for this pH dependence.

Virology studies show that the acidification of vesicles promotes virion maturation for PV (39). Another picornavirus, enterovirus D68 (EV-D68), has also been shown to require an acidic compartment to form RO and produce viral RNA normally (40). In addition, the amount of two other membrane-binding proteins, EV-D68 2B and 3A, decreases upon de-acidification (40). It was speculated that the failure to acidify the replication organelle/assembly organelle compartment could destabilize 2B and 3A, perhaps by decreasing their interaction with membranes and causing their degradation in the cytoplasm (41). Although 2B and 3A of PV do not show this pH sensitivity (40), other PV proteins (*i.e.,* 3C) may have stronger membrane interactions at lower pH.

Our previous unpublished work suggested differences in how untagged and C-terminal hexahistidine-tagged 3C interact with phosphoinositide lipids. A recent study on the binding of HIV-1 matrix protein (MA) and PI(4,5) P2-enriched membranes shows that His-tag can significantly influence MA membrane binding properties, promoting protein–membrane binding and PI(4,5)P_2_ clustering (31). They speculated that the reason derives from the electrostatic nature of the interaction, and they suggested that researchers in this field should be aware of this potential artifact. In our study, according to the supported lipid bilayer assays, His-tagged 3C had a lower dissociation constant than untagged 3C. The presence of the His-tag does not cause a significant conformational change on 3C (Fig. S10), but because His-tagged 3C is more positively charged, it may interact more strongly with the negatively charged membrane. However, the PRE experiments do not show obvious differences in the membrane binding mode of His-tagged 3C and untagged 3C. These studies suggest that while the His-tag may make a small contribution to binding affinity, it does not appear to be the driving factor for 3C-membrane interaction. The smaller surface area of nanodiscs, the different membrane fluidity (42) or interactions with the scaffold protein may be responsible for the difference between the model membrane systems.

Through these studies, we have developed an assay to probe 3C-lipid membranes interactions at an atomically resolved level and have begun to uncover the molecular determinants of these binding interactions. This assay can be leveraged to gain insight into additional factors, including lipid composition and the effect of divalent counterions (43, 44, 45), on 3C-membrane interactions, and can be extended to studying protein-membrane interactions of other viral proteins, including 3D and 3CD. For example, 3CD is more abundant, and the electrostatic potential map of 3CD (Fig. S11) shows that there is also a positively charged surface on the 3D domain of 3CD that is close to the 3C membrane-binding region, which 3CD may leverage in its interactions with replication membranes.

By determining more atomic and physical details of the PV protein-membrane interactions, we will gain insight into how membrane binding alters the conformation, dynamics, interactions, and function of these proteins, enabling a better understanding of the importance of the phosphoinositide-enriched membranous structures that form during positive-strand RNA virus infection.

## Experimental procedures

### Membrane scaffold protein expression and purification

The expression and purification of MSP1D1 was adapted from Ritchie *et al.* (46). The plasmid pMSP1D1 (#20061) was purchased from Addgene and transformed into *E.coli* BL21(DE3) cells and plated on Lysogeny broth (LB) agar plates with 50 μg/ml kanamycin. A single colony was used to inoculate 50 ml NZCYM media with 50 μg/ml kanamycin and shaken at 37 °C, 200 to 250 rpm overnight. A large volume of media (500 ml of NZCYM media with 50 μg/ml kanamycin) was inoculated with 10 ml of the overnight culture and shaken at 37 °C, 250 rpm until the optical density at 600 nm (OD600) reached 0.6 to 0.8, at which time protein expression was induced with 1 mM isopropyl β-D-1-thiogalactopyranoside (IPTG). After 4 h at 37 °C, cells were collected by centrifugation and the resulting cell pellet was frozen at −80 °C. The pellet was thawed on ice, resuspended in 20 mM potassium phosphate pH 7.4, 1% Triton-x100, and lysed by sonication. After centrifugation, 10 mM imidazole was added to the supernatant and MSP1D1 was purified by Ni-NTA chromatography. After loading onto the column, the lysate was washed with 40 ml of the following: 40 mM Tris pH 8, 300 mM NaCl, 1% v/v Triton-x100; 40 mM Tris pH 8, 300 mM NaCl, 20 mM imidazole, 50 mM sodium cholate; 40 mM Tris pH 8, 300 mM NaCl, 50 mM imidazole; 40 mM Tris pH 8, 300 mM NaCl, 400 mM imidazole. Fractions containing MSP1D1, determined by SDS-PAGE, were pooled and dialyzed twice at 4 °C against 2 L of 20 mM Tris pH 7.4, 100 mM NaCl, 5 mM EDTA using 3 to 8 kDa MWCO (molecular weight cut-off) Spectra/Por dialysis tubing. After dialysis, the protein was concentrated to 2.67 mg/ml, determined by measuring the absorbance at 280 nm (ε280 = 21,000 M^−1^ cm^−1^), using a five or 10 kDa MWCO Sartorius Vivaspin spin concentrator. The protein solution was filtered through a 0.2 μm syringe filter, sodium azide was added to 0.01%, and the solution was frozen at −20 °C.

### Lipid nanodisc preparation

Lipid nanodiscs were prepared based on a modified protocol from Bayburt *et al.* (22). All lipids were purchased from Avanti Polar Lipids. Four milligrams of total lipids dissolved in chloroform were dried under nitrogen in a glass vial while gently swirling to ensure even distribution of lipids. After the chloroform was evaporated, the lipids were dried for 4 h in a vacuum desiccator to remove residual solvent. Following this step, 94 μl of 20 mM Tris pH 7.4, 200 mM sodium cholate was added to the dried lipids and vortexed for 2 min to resuspend the lipids. The suspension was placed on ice for 30 min and mixed by flicking every 10 min. Afterwards, 1 ml of 2 mg/ml membrane scaffold protein 1D1 (MSP1D1)-His in 20 mM Tris pH 7.4, 100 mM NaCl, 0.375 mM EDTA, 50 mM sodium cholate was added to the lipid suspension and gently mixed. All nanodiscs used here used MSP1D1-His as the scaffold protein. After 1 h on ice, the lipid-protein suspension was dialyzed at 4 °C against 1 L 20 mM Tris pH 7.4, 100 mM NaCl for 48 h using a 3 to 8 kDa MWCO dialysis membrane with three changes of buffer. After dialysis, the nanodisc mixture was purified by size exclusion chromatography using a Superose 6 Increase 10/300 column in a GE ÄKTA Pure 150L FPLC system. Samples were filtered using a 0.22 μm filter and then injected into the sample loop. The column was run at 0.5 ml/min with 20 mM Tris pH 7.4, 100 mM NaCl while monitoring absorbance at 280 nm and 215 nm. The nanodisc samples for small-angle X-ray scattering and ^31^P NMR were purified by size exclusion multi-angle light scattering (SEC-MALS) using a Wyatt S200 column in an Agilent 1260 Infinity II Liquid Chromatography System, with multi-angle light scattering detected by a Wyatt Dawn detector. The column was run with 20 mM Tris pH 7.4, 100 mM NaCl while monitoring absorbance at 280 nm. The size of the purified nanodiscs was then characterized by dynamic light scattering (DLS) in Wyatt DynaPro NanoStar (data not shown). Nanodisc concentration is determined by measuring the absorbance at 280 nm (ε280 = 42,000 M^−1^ cm^−1^).

### Transmission electron microscopy (TEM)

Purified nanodiscs from FPLC-SEC were diluted with 20 mM Tris pH 7.4. Five microliters of nanodiscs sample were transferred to glow-discharged 400 mesh Carbon Type-B copper grids (Ted Pella). After 1 min incubation, the excess sample was removed with filter paper by side blotting. Five microliters of 2% w/v uranyl acetate solution was applied to the grid and was removed after 30 s by side blotting. Samples were air-dried for at least 5 min. The microstructures of the samples were observed by a FEI Talos F200x transmission electron microscope at 200 kV.

### Small-angle X-ray scattering (SAXS)

Small-angle X-ray scattering was performed using the Rigaku BioSAXS-2000 2D-Kratky system. After collecting 200 μl fractions from SEC-MALS, the fraction with the largest scattering amplitude was used for SAXS. Each scattering profile was measured for 10 min, and repeated 6 times. Three 60 μl volume replicates of the sample and buffer (from SEC-MALS fractions with no scattering or absorbance at 280 nm) were averaged after checking for radiation damage, and the buffer scattering profile was subtracted. The scattering profiles were analyzed using the ATSAS software package (47).

### Expression and purification of poliovirus 3C

^15^N labeled nonHis-tagged poliovirus 3C and His-tagged poliovirus 3C were expressed and purified as previously described (48). For His-tagged 3C, *E.coli* BL21(DE3) PCG1 cells, which express a protease to cleave the N-terminal SUMO tag, were used instead of *E.coli* BL21(DE3) pRARE and only the first Ni-NTA column was used in the purification. The amino acid sequence of nonHis-tagged 3C is based on the wild-type Type 1 Mahoney sequence, generated by cleavage of an N-terminal His-SUMO tag. His-tagged 3C has a C-terminal hexahistidine tag preceded by a GSSG linker. Both variants of 3C had the amino acid substitutions C147A/C153S to avoid oxidation of cysteine residues and inactivate protease activity, as has been used previously (8, 25).

Unlabeled poliovirus 3C proteins were expressed as described for MSP1D1 with the following modifications: Media contained 30 μg/ml chloramphenicol in addition to 50 μg/ml kanamycin. For nonHis-tagged 3C, *E.coli* BL21(DE3) pRARE cells were used. For His-tagged 3C, *E.coli* BL21(DE3) PCG1 cells (49) were used. Unlabeled His-tagged 3C was purified the same as the ^15^N-labeled His-tagged 3C as described above, and unlabeled nonHis-tagged 3C were purified the same as the ^15^N-labeled nonHis-tagged 3C described above. After the final Ni-NTA column, the proteins were concentrated using 10 kDa MWCO Sartorius Vivaspin spin concentrators and dialyzed twice at 4 °C against 2 L of 20 mM HEPES pH 7, 100 mM NaCl.

### Supported lipid bilayer binding assays

Supported lipid bilayers were prepared in microfluidic devices as previously described (3, 8). Small unilamellar vesicles were prepared with the following mole percentages of lipids: 92.3% POPC, 7.5% PI4P (brain), 0.2% ortho-sulforhodamine B-POPE (oSRB). POPC and PI4P were purchased from Avanti Polar Lipids, and oSRB was prepared as previously described (8). After rinsing the microfluidic channels with buffer, 3C-containing solution ranging from 0.5 to 50 μΜ was flowed through for at least 30 min until the fluorescence intensity remained constant. Fluorescence was measured using an Andor iKon-M camera through a Nikon Eclipse Ti-V microscope. The fluorescence of channels containing 3C was analyzed using NIS-Elements software. The fluorescence in each channel was subtracted from the fluorescence of a reference channel in the same microfluidic device.

For nonHis-tagged 3C and His-tagged 3C, three binding curves were fit independently and the resulting dissociation constants were averaged and uncertainty was determined as the standard deviation. The dissociation constant K_d_ was calculated by fitting equation (Equation 1) for K_d_ and ΔF_max_ using least squares minimization (curve_fit function from the optimize module in SciPy v. 1.5.0 in Python 3.8), where ΔF is the difference in fluorescence between the channel containing 3C and the reference channel with no 3C, ΔF_max_ is ΔF at saturating concentration of 3C, [3C] is the concentration of 3C, and K_d_ is the dissociation constant:

(Equation 1) $$\Delta F={\Delta F}_{\max}\frac{\left[3C\right]}{\left[3C\right]+K_{d}}$$

### NMR spectroscopy and PRE experiments

Samples were prepared for NMR PRE experiments by dialyzing purified poliovirus 3C twice against NMR buffer, which contains 20 mM Tris at different pHs (6.5,7, or 7.5) and different NaCl concentrations (60, 80, or 100 mM), and spin concentrating. Nanodiscs for NMR experiments are concentrated using 10 kDa MWCO Amicon Ultra Centrifugal Filter, followed by buffer exchange to NMR buffer *via* diafiltration. The concentrations were 110 μM ^15^N-labeled 3C, 5 μM nanodisc in the NMR buffer containing 10% v/v D_2_O. Nanodiscs contained either 7.5% PI4P, 90% POPC, 2.5% PE-DTPA-Gd^3+^ or 7.5% PI4P, 92.5% POPC. For control experiments, nanodiscs contained either 97.5% POPC, 2.5% PE-DTPA-Gd^3+^ or 100% POPC.

^1^H-^15^N SOFAST HMQC experiments (50) were used to determine intensities of resonances in 3C in the PRE experiment and collect the large number of scans required for reasonable signal-to-noise ratio. A 600 MHz Bruker AVANCE NEO with 5 mm triple resonance (^1^H/^13^C/^15^N/^2^H) Z-gradient TCI cryoprobe was used with the following parameters: 64 scans, 128 indirect points, indirect spectral width of 36 ppm, indirect center frequency 116 ppm, interscan delay of 200 ms. The shaped pulse for selective ^1^H excitation was centered at 8.5 ppm with a spectral width of 4 ppm.

For all experiments, resonance intensities were determined by picking peaks in NMRPipe (51) for a reference spectrum, finding the intensity at the local maximum in each spectrum closest to the reference peak, and manually inspecting each 1D slice to ensure proper peak picking. Resonance assignments are from Amero *et al.* (BMRB Entry 15222) (25). The intensity ratio was calculated by dividing the intensity values for the same peak in each spectrum. Error bars in the PRE plots were calculated by taking the square root of sum of squares of the percent error due to noise from each spectrum being compared and multiplying by the intensity ratio. To calculate the percent error for each peak intensity in a given spectrum, the noise level in the spectrum was divided by the peak intensity. Noise was calculated as the standard deviation of the intensity across 700 points in the ^1^H dimension for a region of the spectrum with no peaks.

^31^P NMR Spectroscopy was performed on a 500 MHz Bruker Avance III-HD spectrometer with a Prodigy BBO cryoprobe. The spectral width was 99.57 ppm, 326,078 points were collected, the recycle delay was 30 s, and 1064 scans were collected, with a total experiment time of 11 h ^1^H decoupling was applied only during acquisition to avoid signal enhancement by NOEs and allow for quantitative integration (pulse program zgig from Bruker Topspin). The nanodisc concentration was ∼50 μM in 20 mM Tris pH 7.4, 100 mM NaCl, 10% D_2_O at a temperature of 298 K with a lipid composition of 96% POPC, 4% PI4P.

### Electrostatic potential map

The electrostatic potential surface calculations were performed using the APBS-PDB2PQR web server (52). Results were visualized with PyMOL.

### Multiple sequence alignments and bioinformatic analysis

3C sequences of Enterovirus prototype strains were aligned by Clustal Omega (53) and then processed by ESPript 3.0 (54) as previously described (8).

## Data availability

All data have been presented in the manuscript and Supporting information.

## Supporting information

This article contains Supporting information (8).

## Conflict of interest

The authors declare that they have no conflicts of interest with the contents of this article.
