# Supplementary material for: Molecular determinants of picornavirus 3C protease binding to phosphoinositide-enriched lipid membranes
Source: J Biol Chem. 2025 Jul 30;301(9):110543. doi: 10.1016/j.jbc.2025.110543 (PMC12446773; doi:10.1016/j.jbc.2025.110543)
Supplement: Supporting Figures [file mmc1.pdf]

Supporting information for

Molecular determinants of picornavirus 3C protease binding to phosphoinositide-enriched lipid membranes

Jie Yu, Dennis S. Winston and David D. Boehr

Department of Chemistry, The Pennsylvania State University, University Park, PA, 16802

This file includes:

Figure S1. Purification of lipid nanodiscs through size-exclusion chromatography.

Figure S2. PV 3C binding to lipid nanodiscs requires PI4P.

Table S1. Conditions, means and standard deviations for PRE experiments.

Figure S3. Collection of PRE results collected under different conditions of pH, [NaCl] and temperature.

Figure S4. Effects of temperature on 3C-nanodisc interaction.

Figure S5. Comparison of 3C-membrane interactions using 3C constructs without and with C-terminal hexahistidine tags (3C-CHis).

Figure S6. Binding curves from supported lipid bilayer binding assays for 3C and 3C with C-terminal hexahistidine tags.

Figure S7. Multiple-sequence alignment of enteroviral 3C proteins.

Figure S8. The PRE results largely agree with previous chemical shift perturbation in the lipid titration experiments.

Figure S9. NMR spectra of 3C at different pH.

Figure S10. NMR spectra of 3C constructs without and with C-terminal hexahistidine tags.

Figure S11. Electrostatic potential map of the surface area of PV 3CD protein.

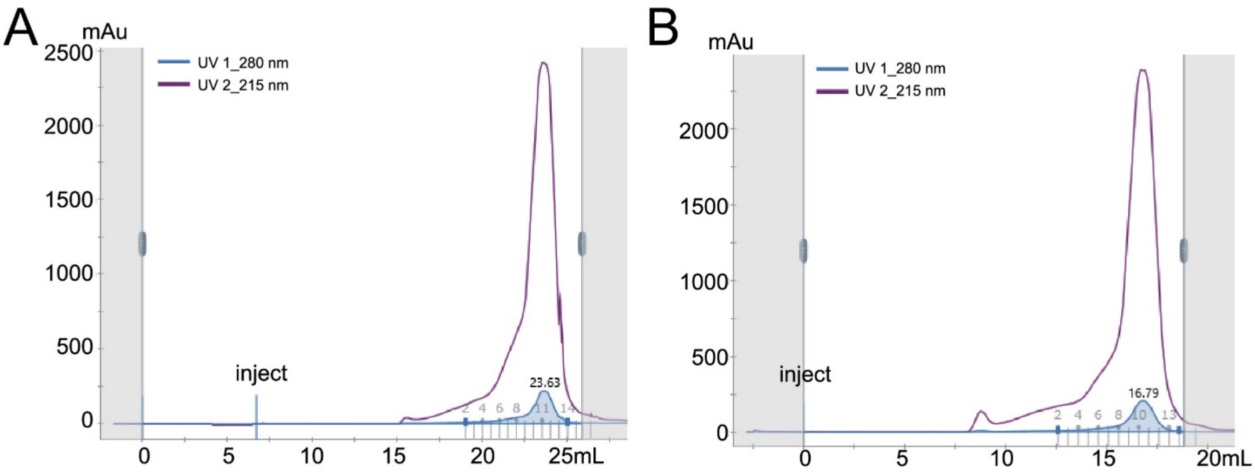

Figure S1. Purification of lipid nanodiscs through size-exclusion chromatography. Nanodisc mixtures were purified by size exclusion chromatography using a Superose 6 Increase 10/300 column in a GE ÄKTA Pure 150L FPLC system. Samples were filtered using a 0.22 µm filter and then injected into the sample loop. The column was run at 0.5 mL/min with 20 mM Tris pH 7.4, 100 mM NaCl while monitoring absorbance at 280 nm and 215 nm. Chromatogram traces for 7.5% PI4P, 92.5% POPC (*A*) and 2.5% PE-DTPA-Gd^{3+} and 7.5% PI4P, 90% POPC (*B*) nanodiscs are shown. Nanodiscs elute at 34min (17mL).

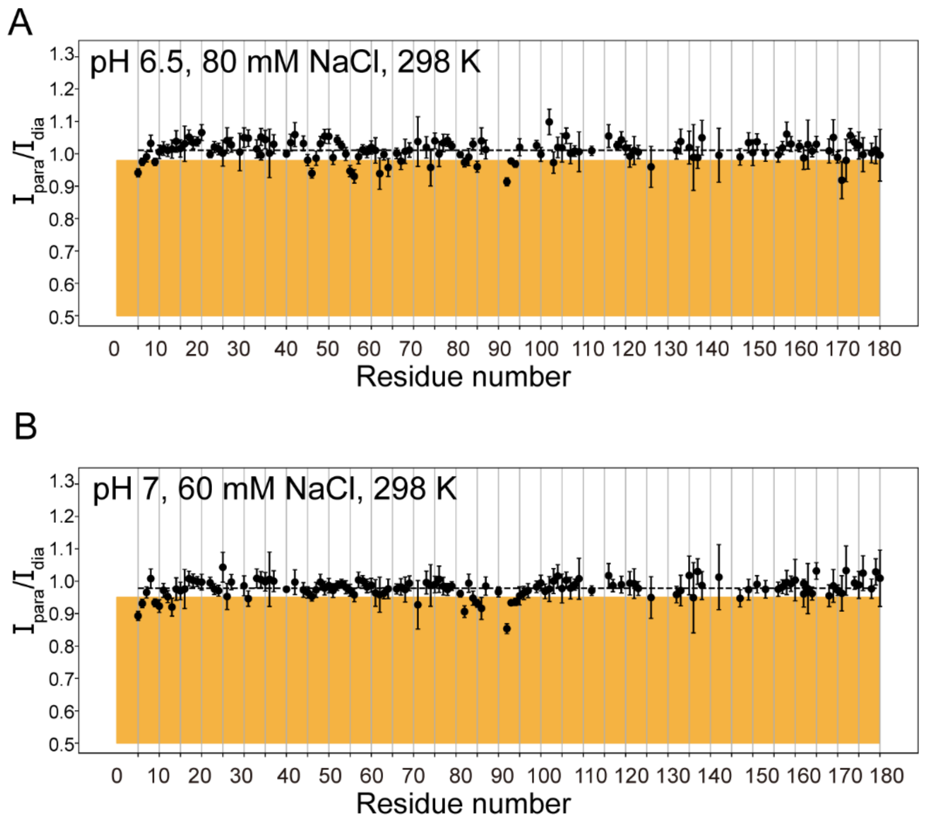

Figure S2. PV 3C binding to lipid nanodiscs requires PI4P. (A) Intensity of resonances for control paramagnetic sample without PI4P (5 µM 2.5% PE-DTPA-Gd^{3+}, 97.5% POPC nanodiscs, 110 µM 3C-CHis in 20 mM HEPES pH 6.5, 80 mM NaCl, 10% D_2O, 298 K) divided intensity for diamagnetic sample (5 µM 100% POPC nanodiscs, 110 µM 3C-CHis in 20 mM HEPES pH 6.5, 80 mM NaCl, 10% D_2O, 298 K) from SOFAST ^1H-^{15}N HMQC spectra. **(B)** Same experiments under pH 7, 60 mM NaCl, 298 K. Error bars reflect noise levels of the spectra.

Table S1. Conditions, means and standard deviations for PRE experiments.

Sample	pH	[NaCl] (mM)	Temperature (K)	Mean	Standard deviation
A	6.5	60	298	1.035	0.066
B	6.5	80	298	0.961	0.045
C	6.5	100	298	0.889	0.034
D	7	60	298	0.913	0.053
E	7	80	298	0.989	0.042
F	7	100	298	0.988	0.034
G	7.5	60	298	1.019	0.049
H	7.5	80	298	0.922	0.036
I	7.5	100	298	0.990	0.030

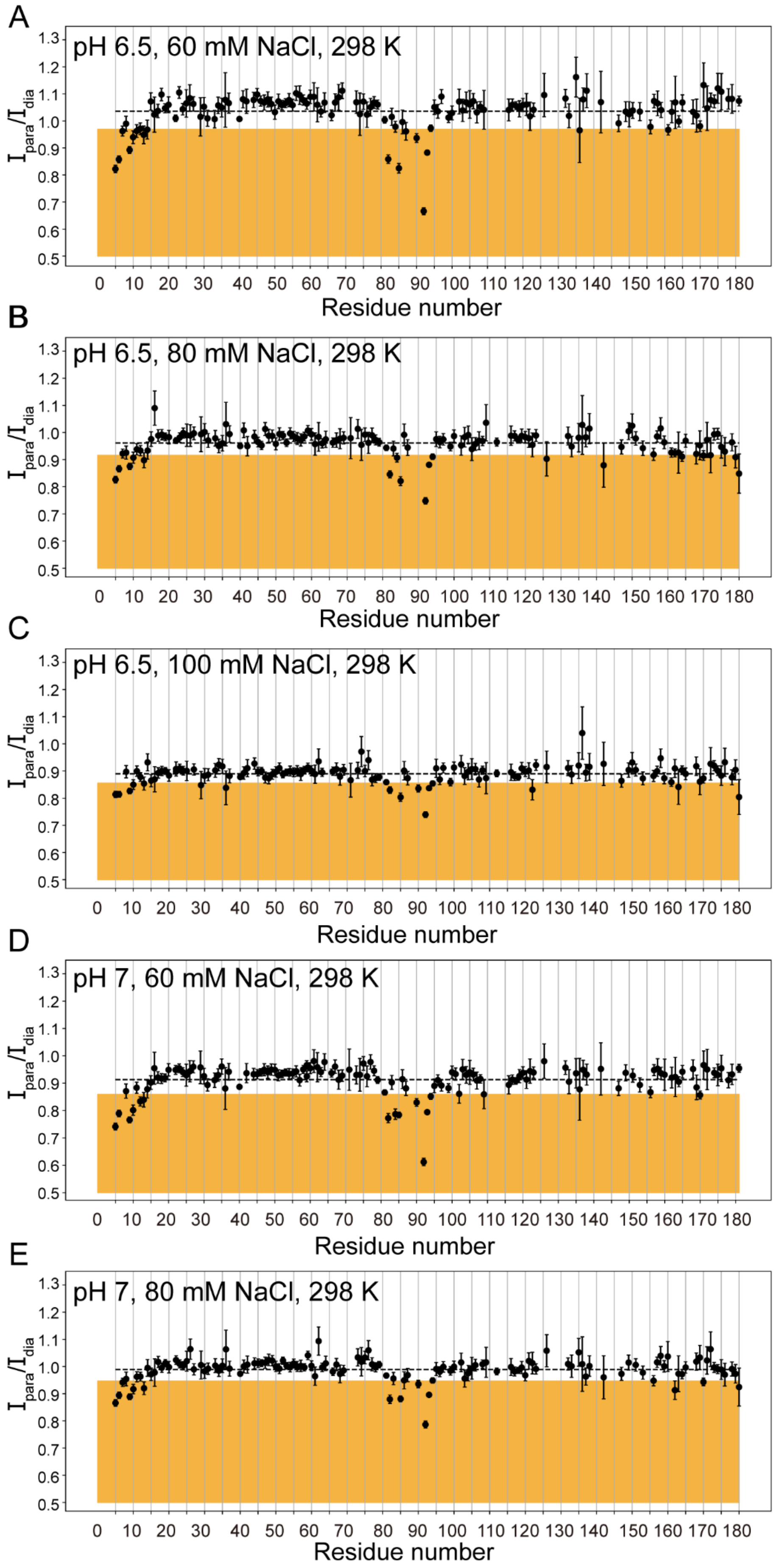
A
pH 6.5, 60 mM NaCl, 298 K
B
pH 6.5, 80 mM NaCl, 298 K
C
pH 6.5, 100 mM NaCl, 298 K
D
pH 7, 60 mM NaCl, 298 K
E
pH 7, 80 mM NaCl, 298 K
I_{para}/I_{dia}
Residue number

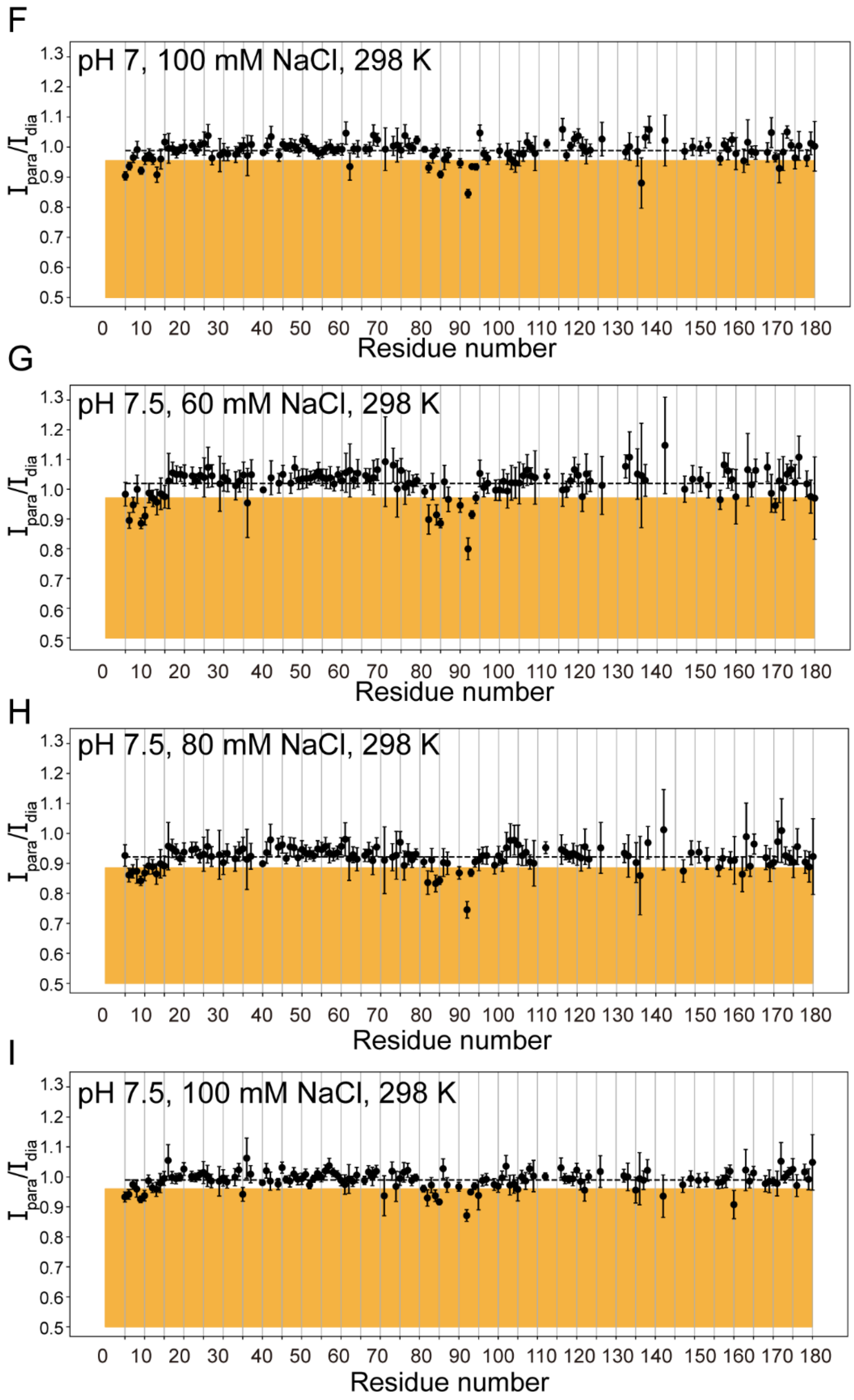

Figure S3. Collection of PRE results collected under different conditions of pH, [NaCl] and temperature. Intensity of resonances for paramagnetic sample (5 μM 2.5% PE-DTPA-Gd^{3+}, 7.5% PI4P, 90% POPC nanodiscs, 110 μM 3C in NMR buffer with 10% D_2O) divided intensity for diamagnetic sample (5 μM 7.5% PI4P, 92.5% POPC nanodiscs, 110 μM 3C in NMR buffer with 10% D_2O) from SOFAST ^1H-^{15}N HMQC spectra. The mean value is shown by a dashed line and the orange background shows residues with intensity ratio one standard deviation below the mean. Error bars

reflect noise levels of the spectra.

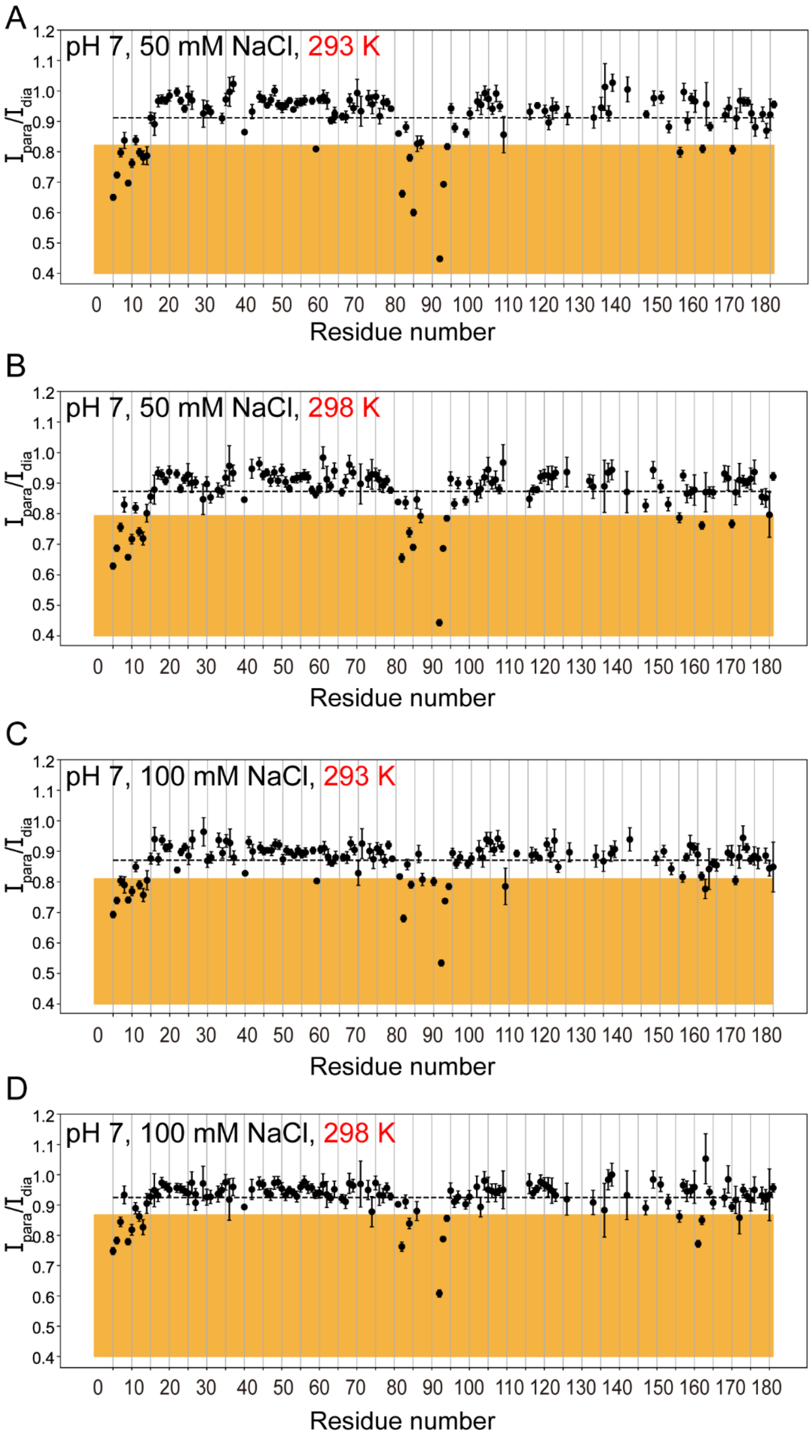

Figure S4. Effects of temperature on 3C-nanodisc interaction. (A-B) Intensity of resonances for paramagnetic sample (**A**: 5 μM 2.5% PE-DTPA-Gd^{3+}, 7.5% PI4P, 90% POPC nanodiscs, 130 μM 3C

in 20 mM HEPES pH 7, 50 mM NaCl, 10% D_2O, 293 K, **B**: 5 μM 2.5% PE-DTPA-Gd^{3+}, 7.5% PI4P, 90% POPC nanodiscs, 130 μM 3C in 20 mM HEPES pH 7, 50 mM NaCl, 10% D_2O, 298 K,) divided intensity for diamagnetic sample (**A**: 5 μM 7.5% PI4P, 92.5% POPC nanodiscs, 130 μM 3C in 20 mM HEPES pH 7, 50 mM NaCl, 10% D_2O, 293 K, **B**: 5 μM 7.5% PI4P, 92.5% POPC nanodiscs, 130 μM 3C in 20 mM HEPES pH 7, 50 mM NaCl, 10% D_2O, 298 K) from SOFAST ^{1}H-^{15}N HMQC spectra. The mean value is shown by a dashed line and the orange background shows residues with intensity ratio one standard deviation below the mean. **(C-D)** Same experiments under pH 7, 100 mM NaCl (C: 293 K, D: 298 K). Error bars reflect noise levels of the spectra.

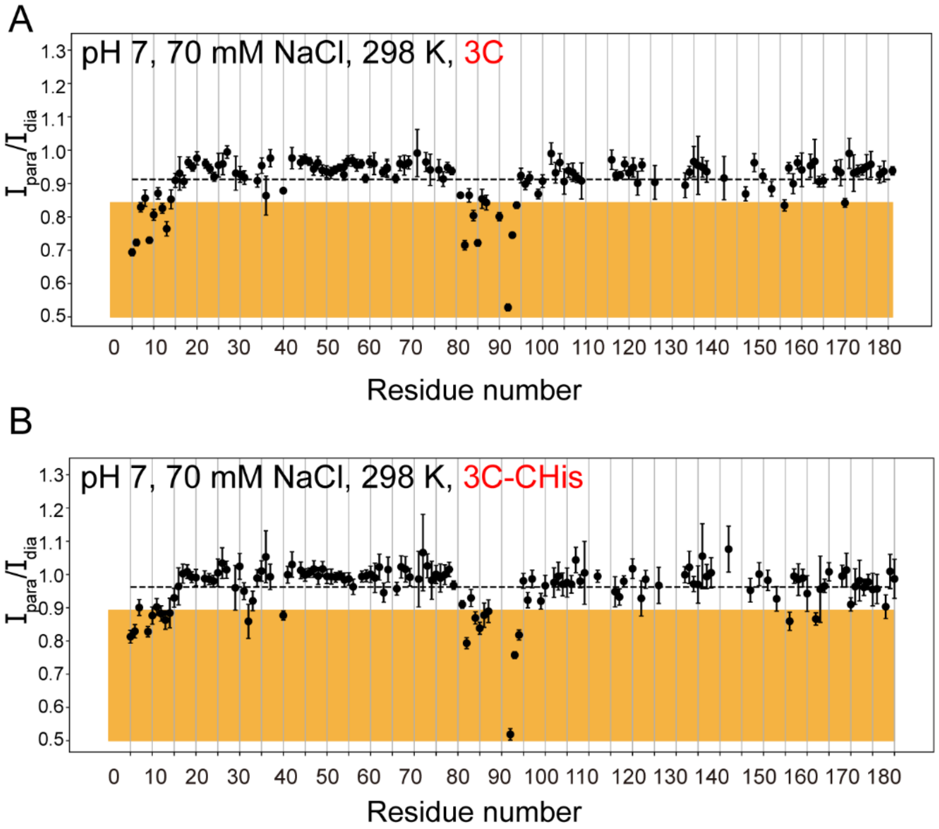

Figure S5. Comparison of 3C-membrane interactions using 3C constructs without and with C-terminal hexahistidine tags (3C-CHis). (A-B) Intensity of resonances for paramagnetic sample (**A**: 5 μM 2.5% PE-DTPA-Gd^{3+}, 7.5% PI4P, 90% POPC nanodiscs, 130 μM 3C in 20 mM HEPES pH 7, 70 mM NaCl, 10% D_2O, **B**: 5 μM 2.5% PE-DTPA-Gd^{3+}, 7.5% PI4P, 90% POPC nanodiscs, 130 μM 3C-CHis in 20 mM HEPES pH 7, 70 mM NaCl, 10% D_2O) divided intensity for diamagnetic sample (**A**: 5 μM 7.5% PI4P, 92.5% POPC nanodiscs, 130 μM 3C in 20 mM HEPES pH 7, 70 mM NaCl, 10% D_2O, **B**: 5 μM 7.5% PI4P, 92.5% POPC nanodiscs, 130 μM 3C-CHis in 20 mM HEPES pH 7, 70 mM NaCl, 10% D_2O) from SOFAST ^1H-^{15}N HMQC spectra. The mean value is shown by a dashed line and the orange background shows residues with intensity ratio one standard deviation below the mean. Error bars reflect noise levels of the spectra.

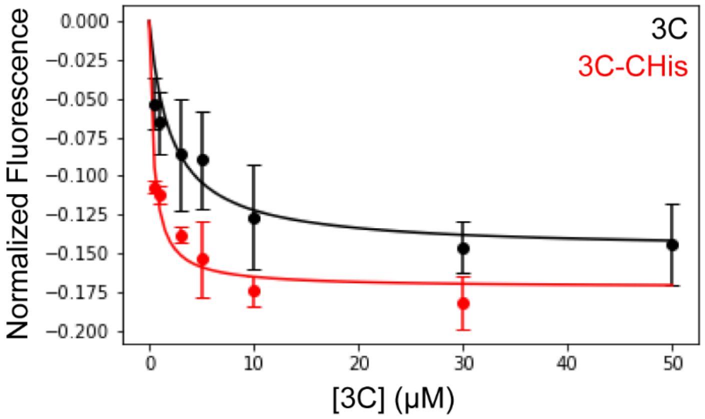

Figure S6. Binding curves from supported lipid bilayer binding assays for 3C and 3C with C-terminal hexahistidine tags. Fitting to the Langmuir adsorption model, dissociation constants were determined to be 2.1 ± 0.9 μM for 3C and 0.41 ± 0.09 μM for 3C-CHis. Three replicates were analyzed using unpaired t test. $p < 0.05$.

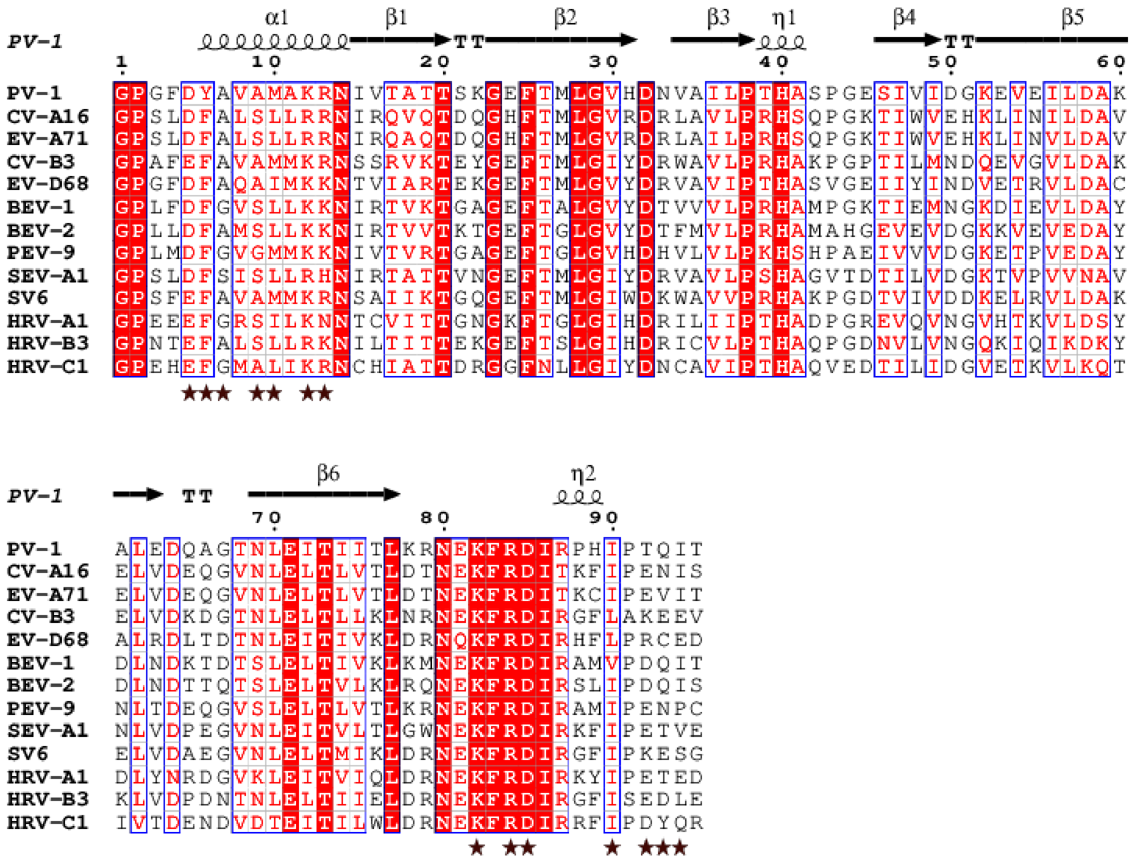

Figure S7. Multiple-sequence alignment of enteroviral 3C proteins. The conserved residues are boxed in blue. Identical conserved and low conserved residues are highlighted in white letters on a red background and red letters, respectively. The residues that are close to the PI4P-enriched lipid membrane according to PRE experiments are labeled using brown stars.

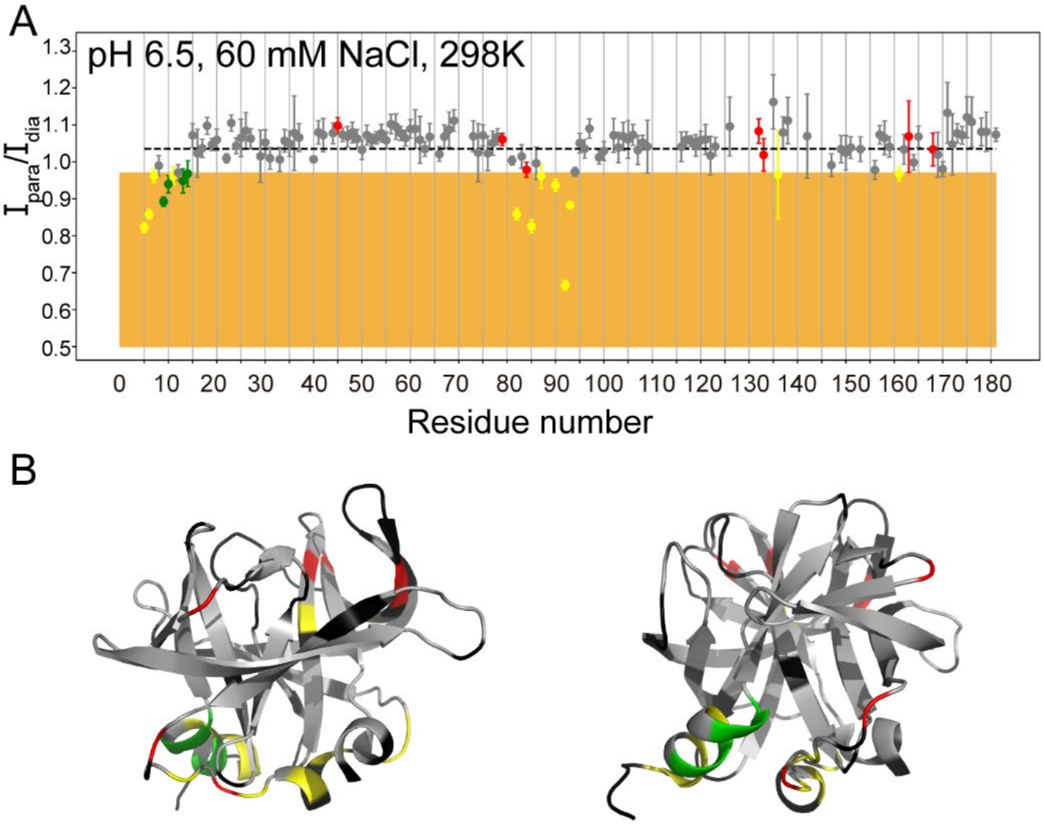

Figure S8. The PRE results largely agree with previous chemical shift perturbation in the lipid titration experiments. (A) PRE data at conditions involving pH 6.5, 60 mM NaCl, 298 K with points colored based on agreement between PRE and lipid titration experiments (taken from ref (1)). Residues were considered close to the membrane in the PRE experiment if they had Ipara/Idia less than one standard deviation below the mean, and close to the membrane in the lipid titration experiments if their chemical shift perturbations were at least one standard deviation above the mean. Residues were colored as follows. Green: close to the membrane in both PRE and lipid titration experiments. Red: close to the membrane in lipid titration experiments but not PRE. Yellow: Close to the membrane in PRE but not lipid titration experiments. Gray: Not close to the membrane in either PRE or lipid titration experiments. Black: no PRE data. **(B)** structure of 3C colored with the same scheme as panel A (pdb 1l1n). Error bars reflect noise levels of NMR spectra.

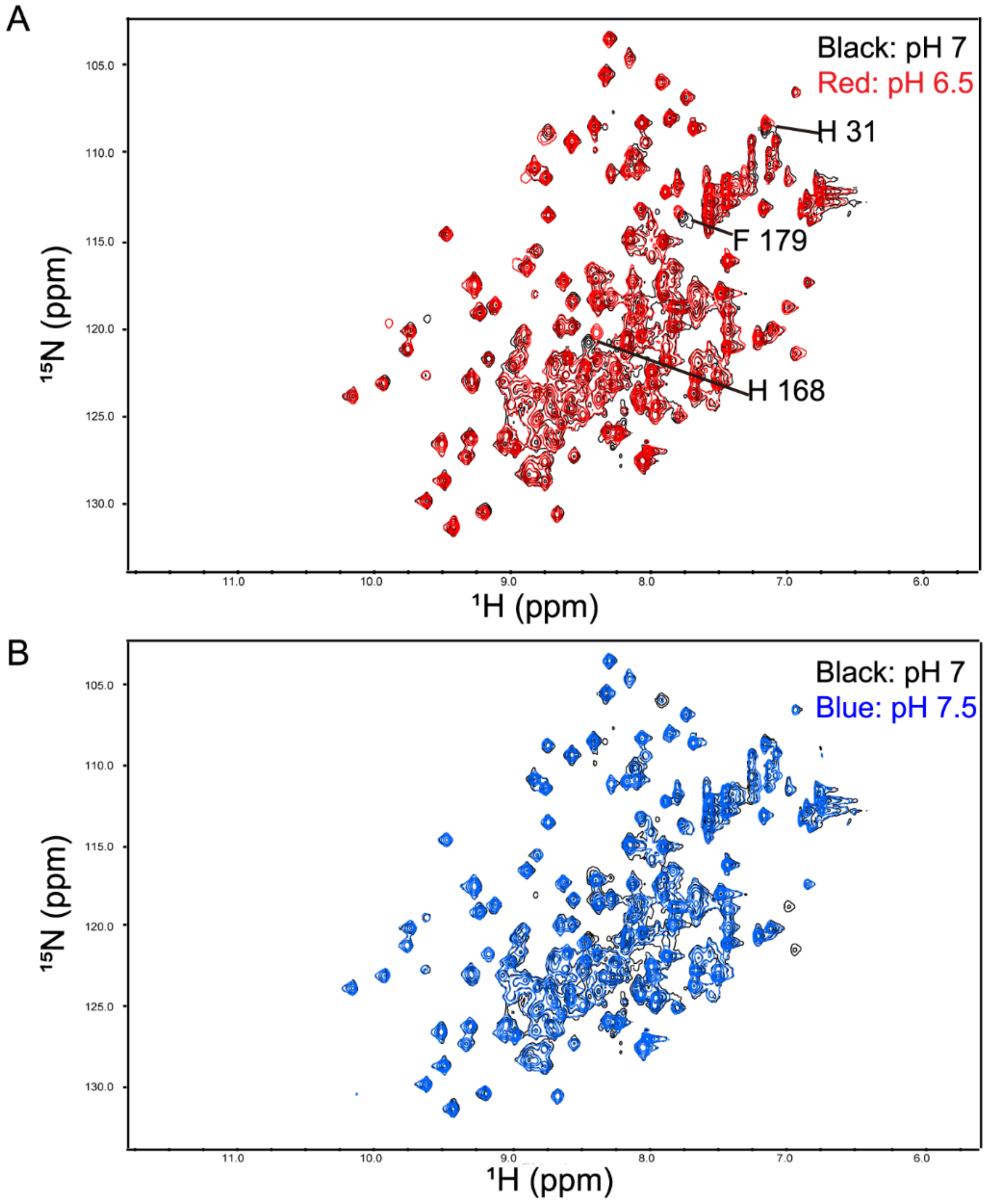

Figure S9. NMR spectra of 3C at different pH. SOFAST ^{1}H-^{15}N HMQC spectra of 110 µM 3C at pH 6.5 (red), 7 (black), and 7.5 (blue) in 20 mM HEPES, 50 mM NaCl, 10% D_2O, 298 K.

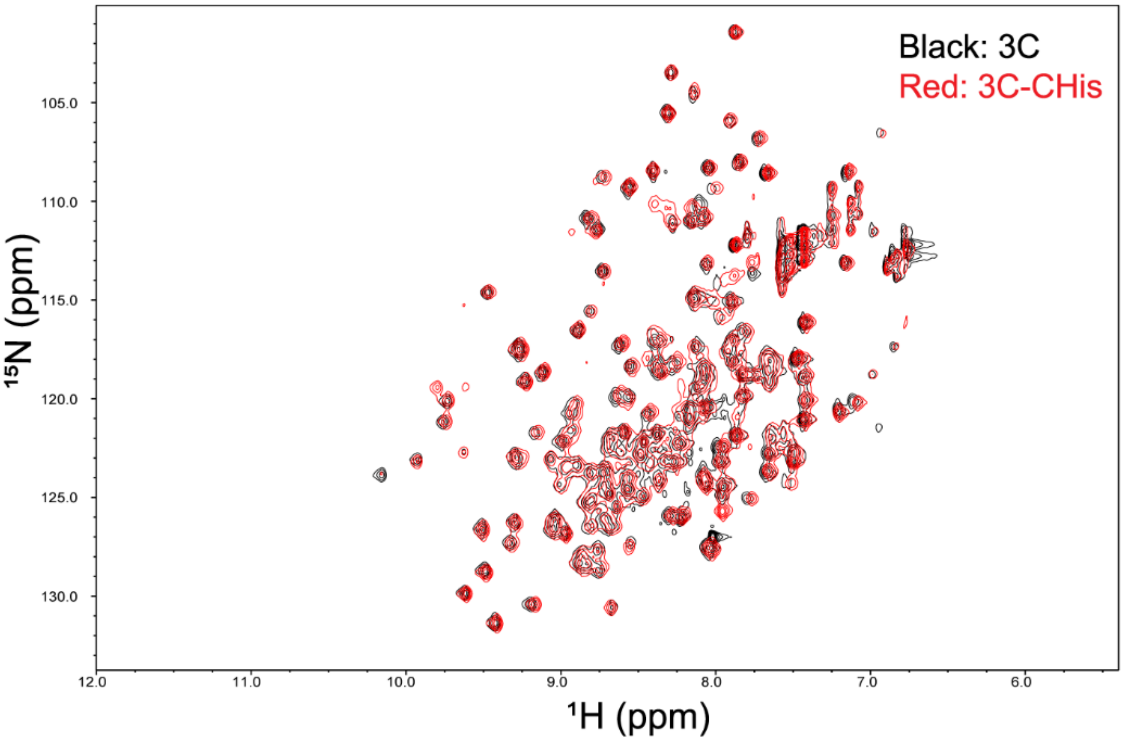

Figure S10. NMR spectra of 3C constructs without and with C-terminal hexahistidine tags. SOFAST ^{1}H-^{15}N HMQC spectra of 110 µM 3C (black) and 3C-CHis (red) in 20 mM HEPES pH 7, 50 mM NaCl, 10% D_2O, 298 K.

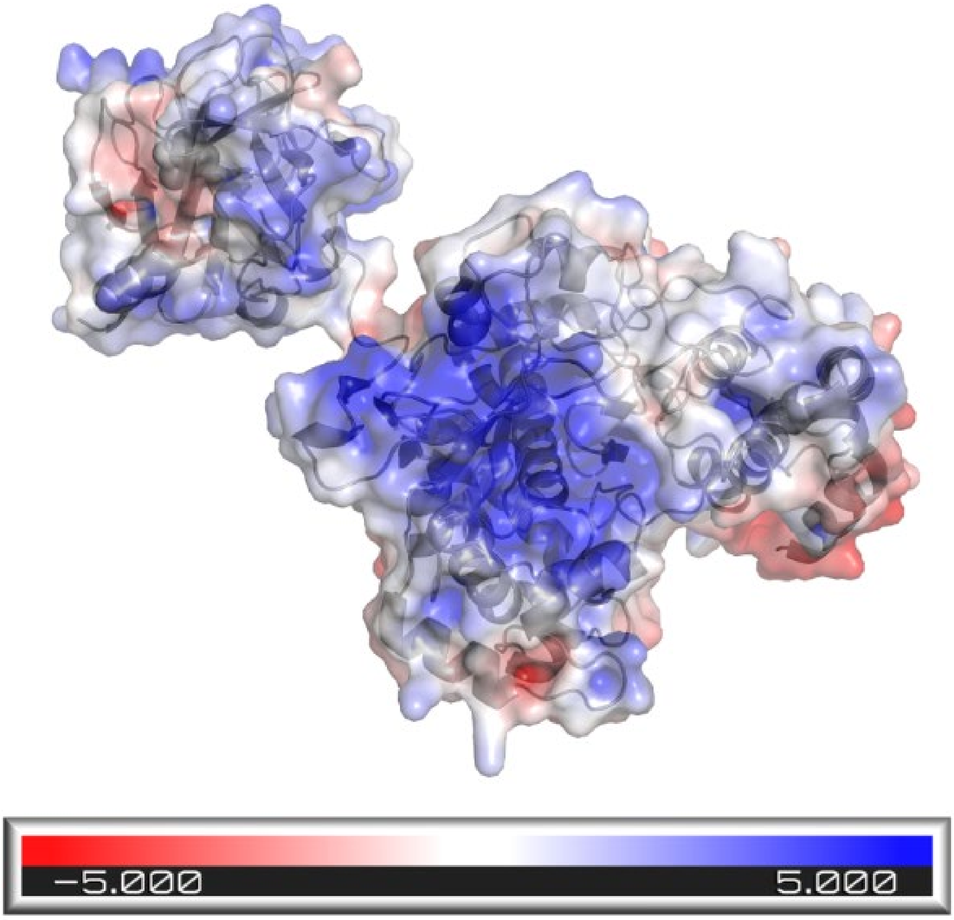

Figure S11. Electrostatic potential map of the surface area of PV 3CD protein. Positive potential is highlighted in blue, negative one in red, neutral regions are shown in white. The protein backbone is colored in dark grey.

References

1. Shengjuler, D., Chan, Y. M., Sun, S., Moustafa, I. M., Li, Z. L., Gohara, D. W., Buck, M., Cremer, P. S., Boehr, D. D., and Cameron, C. E. (2017) The RNA-Binding Site of Poliovirus 3C Protein Doubles as a Phosphoinositide-Binding Domain. *Structure*. **25**, 1875-1886.e7
